# What do parents of school-aged children want to know about HPV vaccination in Canada? Results of an online survey on Facebook

**DOI:** 10.14745/ccdr.v52i03a04

**Published:** 2026-03-31

**Authors:** Mylène Tantchou Dipankui, Benjamin Giguère, Kieran C O’Doherty, Antonella Pucci

**Affiliations:** 1 University of Guelph, Department of Psychology/College of Social and Applied Human Sciences, Guelph, ON; 2 Canadian Public Health Association, Ottawa, ON

**Keywords:** HPV, vaccination, misinformation, Facebook, social media, social marketing, infographics, survey

## Abstract

**Background:**

To identify the source of information, preferred promotional formats, and the type of information parents of school-aged children eligible for human papillomavirus (HPV) vaccination would consider in their decision-making process.

**Methods:**

A bilingual (English and French) pre-campaign survey employing Facebook advertisements was used to recruit parents of school-aged children aged between 9 and 15 years across Canada to participate in the study. The survey consisted of 20 closed-ended and two open-ended questions. Recruitment on Facebook occurred between March 2021 and February 2022. Participants meeting specific requirements were eligible to participate in the study.

**Results:**

A total of 764 parents participated in the study, and 554 met the eligibility criteria. Representation was obtained from nine provinces and two territories. The majority of respondents (24.9%; n=138) indicated that they turned to sources other than social media platforms when it came to making decisions about HPV vaccination. Among other sources, respondents first considered the recommendations made by their healthcare providers (17.5%; n=38). Respondents also reported infographics as the format most likely to influence their decision-making (20.8%; n=115), over other types of resources. Finally, potential side-effects associated with HPV vaccines versus HPV infection outcomes were among the main topics of information the respondents looked at in their decision-making process.

**Conclusion:**

Infographics can be an important social marketing component for educating parents about the vaccination of school-aged children against HPV. These visual representations should focus on HPV vaccine safety, to respond to the information needs of parents. This intervention should be combined with healthcare provider recommendations.

## Introduction

Human papillomavirus (HPV), a sexually transmitted infection, is the cause of several cancers, including cervical cancer ((1–3)). It is estimated that 550,000 Canadians are infected with HPV each year, and almost 80% of females of reproductive age will be infected at some point in their life ((4)). Cervical cancer causes over 400 deaths in Canada per year, and more than 1,300 Canadians are diagnosed each year ((5)). Preventive interventions, such as vaccination and screening, are known to reduce the risk of cervical cancer and death.

The HPV vaccination is part of routine, school-based immunization programs in Canada. The efficacy of the vaccine is optimal (close to 100%) when it is administered before exposure to the targeted HPV types ((6,7)). Provinces and territories in Canada have typically offered a two-dose HPV vaccination program for children in grades 4 through 10 ((8)).

The SARS-CoV-2 pandemic led to a disruption in routine vaccination programs, resulting in many children missing doses of HPV vaccine ((9)). The impact of the pandemic on vaccination coverage rates against HPV for school-aged children has been examined by public health experts. For example, in Ontario, 3.0% of 12-year-old students received HPV vaccine for the 2020–2021 school year ((10)). This proportion increased to 16.6% in 2021–2022 and 47.8% in 2022–2023 ((10)). Before the pandemic, a pooled analysis showed that national vaccine coverage for HPV was approximately 55.91% ((4)). Despite the existing free and nationally available school-based programs and strategies developed across the country to catch up on missing vaccinations, the hesitancy of parents towards HPV vaccination for their children can compromise HPV vaccine uptake ((11)).

The HPV vaccine safety, side-effects, and effectiveness are among the primary concerns of vaccine-hesitant parents ((11–15)). While many parents perceive their healthcare providers to be a key source of information and advice about HPV vaccines ((14,16)), they can also consult online platforms and social networks to search for other information and share their doubts about vaccines ((17)). The downside is that social media and online social networks are the medium for disseminating information that maximizes engagement ((18)), without measures to control the quality of the content shared on such portals ((19)). A well-established pitfall of social media and online social networks is the propagation of misinformation. Following Guess and Lyons ((20)), this study defines misinformation as constituting a claim that contradicts or distorts common understandings of verifiable facts. Such misinformation, when spread on social media, can incite negative emotions towards vaccines, and may lead to an increase in vaccine hesitancy ((21–23)).

In this context, social marketing represents a relevant approach to help address vaccine hesitancy among parents of school-aged children eligible for HPV vaccination ((24)). This approach could assist in designing unique communication strategies for parents, to help increase HPV vaccine uptake ((24)). This project results from collaboration between researchers from the University of Guelph and the Canadian Public Health Association (CPHA). The objective of this research was to identify the preferred promotional formats and type of information that parents of school-aged children eligible for HPV vaccination would consider in their decision-making process to vaccinate their children against HPV. As far as available information indicates, there are no recorded studies examining what Canadian parents want to know about HPV vaccination to inform their decision. Instead, previous studies have primarily focused on parents’ perceptions, knowledge, attitudes and/or behaviours regarding HPV vaccination for their children ((25–29)), and a few on the effects of psychosocial determinants on parental decision-making ((30,31)).

## Methods

### Participant recruitment

The CPHA’s Facebook page was used to display advertisements to recruit a convenience sample of parents of school-aged children on Facebook, over an 11 month period, from March 2021 to February 2022. The advertisements led participants to a Facebook landing page where they consented to participate in the study.

Participants meeting the following requirements were eligible: 1) Must be 18 years of age or older; 2) have at least one child between 9 and 15 years of age in their care; 3) live in Canada; and 4) be able to respond to questions in English or French. A sample of 1,250 parents (n=1,000 for the English sample and n=250 for the French sample) were targeted, based on the participation rates of parents of school-aged children from previous projects. This represents a convenience sample compared to the Canadian population estimated at 36.3 million in 2021 (including citizen and non-citizen) ((32)). All responses were anonymized. Two reminders were sent to non-responders to optimize the response rate.

### Measures

The survey consisted of 20 closed-ended and two open-ended questions and took approximately 15 minutes to complete (see the survey questionnaire in the **Appendix**, **Supplemental material**). Components of the Vaccine Hesitancy Scale (VHS) ((33)) were adapted to the COM-B model for behaviour change ((34,35)), to develop the questionnaire. The VHS has face validity, and its psychometric properties and validity have been assessed in low-income and developed countries ((36,37)). Additionally, adapted versions of the tool have been used in studies across the world, revealing that VHS is a reliable tool for measuring vaccine hesitancy ((38–42)).

The COM-B model ((34,35)) is a behaviour change framework that builds upon 19 behaviour change framework, suggesting that the following three components are important for a behaviour to occur:

1. Capability, which refers to the knowledge, skills and abilities to engage in the behaviour

2. Opportunity, or external factors that make performing the behaviour possible

3. Motivation, or internal processes that influence decision-making and direct the behaviour

The framework’s reliability has been well-established, and it is widely used to develop behavioural interventions. Items specific to social media sources, preferred promotional formats, and the type of information parents would consider in their decision-making were developed in collaboration with Immunize Canada, a national coalition of non-governmental, professional, health, government, and private sector organizations dedicated to promoting the benefits of immunization. This work focuses on HPV information sources, HPV promotional formats, and relevant information that parents of school-aged children, who are eligible for the HPV vaccine would prioritize in their decision-making process. The answers to these questions were crucial for the researchers to implement an effective social marketing campaign.

### Data analysis

The collected data were compiled and analyzed using SPSS software. Data cleaning consisted of checking the data to identify and remove problematic cases. Cases with a 20% or more non-completion rate were deleted. There were 210 respondents were excluded because they did not meet at least one of the eligibility criteria or had a 20% or higher non-completion rate. Variables were checked for univariate outliers. The responses to questions about HPV information sources were grouped into two main variables: 1) social media sources, and 2) “other” sources. Social media sources combined the responses of participants who stated that they relied on Facebook, Twitter, Instagram, Pinterest, and/or Reddit to obtain credible information about vaccines and vaccination for HPV. Other sources combined the responses of participants who relied on scholarly and/or non-scholarly sources that were not considered social media to obtain credible information about vaccines and vaccination for HPV (see **Table 1** for more details). The results were reported with frequency distributions.

**Table 1:** Frequency table of social media and other sources the participants relied on to obtain credible information about human papillomavirus vaccines and vaccination

Social media source	N^a^	Percentage (%)
Other	138	24.9
Facebook	129	23.3
Reddit	43	7.8
Twitter	32	5.8
Pinterest	15	2.7
Instagram	5	0.9
Total	362	65.3
Missing	192	34.7
Total	554	100.0

^a^ Number of respondents who checked the box

## Results

### Participant demographics

Among the 554 parents who met the eligibility criteria and were included in the study, 53.8% of respondents (n=298) had one child between 9 and 15 years of age, while only 2.3% (n=13) had four children between 9 and 15 years of age. Most respondents (92.6%; n=513) were female caregivers or mothers; only 6.3% (n=35) identified as male. Representation was obtained from nine provinces and two territories. These results are summarized in **Table 2**.

**Table 2:** Frequency table of the demographic characteristics of the participants

Demographics characteristics	Sample size (N)	Percentage (%)
**Ages (years)**	**553**	**99.8**
25–34	28	5.1
35–44	317	57.2
45–54	198	35.7
55–64	10	1.8
Missing	1	0.2
**Sex**	**553**	**99.8**
Female	513	92.6
Male	35	6.3
Chose not to respond	5	0.9
Missing	1	0.2
**Gender**	**554**	**100.0**
Woman	508	91.7
Man	36	6.5
Other	3	0.5
Chose not to respond	7	1.3
**Level of education**	**554**	**100.0**
Some high school	6	1.1
High school	32	5.8
Some college or university	64	11.6
College diploma	127	22.9
Apprenticeship training and trades	17	3.1
Professional certification	37	6.6
Undergraduate degree	162	29.2
Graduate degree	109	19.7
**Province of residence**	**552**	**99.6**
Alberta	90	16.2
British Columbia	75	13.5
Manitoba	36	6.5
New Brunswick	21	3.8
Newfoundland and Labrador	6	1.1
Northwest Territories	2	0.4
Nova Scotia	27	4.9
Ontario	194	35.0
Québec	49	8.8
Saskatchewan	48	8.7
Yukon	4	0.7
Total	552	99.6
Missing	2	0.4
**Number of children ages 9–15**	**551**	**99.5**
1	298	53.8
2	197	35.6
3	43	7.8
4	13	2.3
Missing	3	0.5
**Number of children ages 9–15, female**	**548**	**98.9**
0	211	38.1
1	259	46.8
2	70	12.6
3	6	1.1
4	2	0.4
Missing	6	1.1
**Number of children ages 9–15, male**	**548**	**98.9**
0	204	36.8
1	260	46.9
2	71	12.8
3	12	2.2
4	1	0.2
Missing	6	1.1

### Social media and other sources the participants relied on to obtain credible information

A majority of participants reported relying on “other” sources of information to obtain credible information about HPV vaccines and vaccination (24.9%; n=138) (see Table 1). They chose Facebook as the second most used source (23.3%; n=129). Reddit was the third social media source caregivers relied on (7.8%; n=43), with being Twitter (now known as X) being the fourth (5.8%; n=32).

The “other” sources were grouped in **Table 3** into two main categories: scholarly sources and non-scholarly sources. These were sources other than social media that participants specified as influencing their decision-making about HPV vaccines and vaccination. Among respondents who selected the “other” option, 48.4% (n=105) relied on non-scholarly sources, while 17.1% (n=37) relied on scholarly sources (see Table 3). Among non-scholarly sources, parents stated that they relied mainly on information from healthcare providers (17.5%; n=38). Parents also stated that they relied on information from search engines and websites (11.1%; n=24). Only 0.7% (n=4) relied on personal experience to obtain credible information about HPV vaccines and vaccination. Finally, 20.3% (n=44) of parents stated that they do not consult social media for vaccine information. These results are summarized in **Table 4**.

**Table 3:** Frequency table of “other” sources the participants relied on to obtain credible information about human papillomavirus vaccines and vaccination

Source	N	Percent (%)	Valid percent^a^ (%)
Non-scholarly sources	105	19.0	48.4
Non-classifiable	75	13.5	34.6
Scholarly sources	37	6.7	17.1
Total	217	39.2	100.0
Missing	337	60.8	N/A
Grand total	554	100.0	N/A

Abbreviation: N/A, not applicable

^a^ Percentage after missing responses are disregarded

**Table 4:** Frequency table of non-scholarly sources parents relied on to obtain credible information about human papillomavirus vaccines and vaccination

Sources	N	Percent (%)	Valid percent^a^ (%)
Don’t consult social media for vaccine information	44	7.9	20.3
Healthcare providers	38	6.9	17.5
Scholarly sources	37	6.7	17.1
Other	31	5.6	14.3
Search engines and websites	24	4.3	11.1
News and videos	16	2.9	7.4
Public health organizations	14	2.5	6.5
Personal experience	4	0.7	1.8
Blogs	1	0.2	0.5
Applications	7	1.3	3.2
Drug label	1	0.2	0.5
Total	217	39.2	100.0
Missing	337	60.8	N/A
Grand Total	554	100.0	N/A

Abbreviation: N/A, not applicable

^a^ Percentage after missing responses are disregarded

### Promotional formats used to provide human papillomavirus vaccination information that could influence parents’ decision-making

Infographics were reported as the format most likely to influence a participant’s decision-making (n=115); other sources (e.g., information from healthcare professionals and public health organizations, and personal experience) ranked second (n=86), narratives/stories (n=67) ranked third, and videos ranked fourth (n=50). Images were ranked fifth (n=19) (see **Table 5**).

**Table 5:** Frequency table of the promotional formats used to provide human papillomavirus vaccination information, in order from most likely “1” to least likely “5” to influence decision-making

Promotional formats	N^a^	Percent (%)	Valid percent^b^ (%)
Infographics	115	20.8	34.1
Others	86	15.5	25.5
Narratives/stories	67	12.1	19.9
Videos	50	9.0	14.8
Images	19	3.4	5.6
Total	337	60.8	100.0
Missing	217	39.2	N/A
Grand total	554	100.0	N/A

Abbreviation: N/A, not applicable

^a^ Number of participants ranking the format first

^b^ Percentage after missing responses are disregarded

### Information that parents considered to make their decision about human papillomavirus vaccination

Side-effects of HPV vaccines was the main information topic parents searched for when making decisions about HPV vaccination (359 respondents selected this option); HPV infection outcomes (e.g., cancer) followed (263 respondents). Information on the chances of getting HPV infection (233 respondents) and how HPV vaccines are tested (203 respondents) was also important. Homeopathic remedies (29 respondents) and alternative HPV vaccination schedules (61 respondents) were reported as among the least valuable information (see **Table 6** for the results and the predefined responses). The main findings of this study are summarized in the Supplemental material.

**Table 6:** Information that parents looked for to make their decision about human papillomavirus vaccination

Information parents looked for in order to make their decision about HPV vaccination	N^a^
What are the side-effects of HPV vaccines?	359
HPV infection outcomes (e.g., cancer)	263
Chances of getting HPV infection	233
How are HPV vaccines tested?	203
Who regulates HPV vaccine safety?	190
Who monitors HPV vaccine safety?	185
Is it safe to get more than one vaccine at the same time?	170
What are HPV vaccine ingredients?	169
Can someone get sick from an HPV vaccine?	139
Who is providing the vaccine (e.g., doctor, nurse, etc.)?	136
Are additives in HPV vaccines safe?	135
How is the HPV vaccination schedule tested?	104
How to minimize HPV vaccine injection pain and fear?	82
Alternative HPV vaccination schedules	61
Other	33
Homeopathic remedies	29
Don’t know/Not sure	21

Abbreviation: HPV, human papillomavirus

^a^ Number of participants who checked the box

## Discussion

This study was conducted to identify the source of information, the preferred promotional formats, and type of information parents of school-aged children eligible for HPV vaccination would consider in their decision-making process.

The study revealed that parents (respondents) relied first on sources other than social media to obtain credible information about HPV vaccines and vaccination. They considered their healthcare providers to be the most important source of information in influencing their decision-making around HPV vaccines and vaccination for their children. These results confirmed that, on average, parents value the information and recommendations provided by their children’s healthcare providers ((43)) more than what is posted on social media. Therefore, healthcare providers may be crucial in improving HPV vaccination uptake rates among school-aged children ((44)). The impact of this result can be twofold. First, when healthcare providers are concerned about parents’ potential negative reactions to an HPV vaccination recommendation, these findings should provide reassurance ((43)). At the same time, healthcare providers will need to be prepared to provide accurate information on HPV vaccination to both pro-vaccine and vaccine-hesitant parents ((43)). Second, a healthcare provider’s vaccine concerns, hesitancy, and inadequate knowledge could hinder them from encouraging parents to get their children vaccinated ((44)). Public health organizations should ensure that healthcare providers have the support and education they need to be informed about HPV vaccination, to reduce any vaccine hesitancy that they themselves may have ((44)).

Parents also reported infographics as the promotional format most likely to influence their decision-making regarding HPV vaccines and vaccination. An infographic is defined as a visual representation of educational content ((45)). Infographics are commonly used to deliver complex information, communicate scientific facts, and foster behavioural change ((45)). When designed properly, infographics can engage both specialists and laypersons ((46)). Li *et al.* (46) suggest that an infographic that is properly designed can illustrate concepts, clarify data patterns, and provide aesthetic pleasure. Research shows that visually simple infographics, when the visual message presents orderliness, balance, and clarity, are more effective in driving behavioural change, compared to messages that are complex ((46)). For example, in a large longitudinal study involving young adults, Garcia-Retamero *et al.* ((47)), conducted an eight-hour educational intervention. They examined the impact of the intervention on the efficacy of a message for promoting condom use. The authors noted that simple brochures featuring visual aids changed attitudes and behavioural intentions as effectively as an extensive intervention. These findings suggest the importance of making the visual message simple when designing infographics on HPV vaccination and countering manipulative and false messages.

Finally, side-effects of HPV vaccines were the main information topic that parents sought when making their decision about HPV vaccination. These results align with previous work. For example, a study from the United States National Cancer Institute showed an increase in the percentage of parents who declined the HPV vaccine for their children due to safety concerns, from 13% in 2015 to 23% in 2018. However, reports of serious health issues after HPV vaccination were consistently rare during the same period ((48)). The authors hypothesize that rising vaccine safety concerns among parents drive increased social media use and parents’ reliance on online sources to find vaccine information. Therefore, it might be relevant to address parents’ concerns regarding vaccine safety online.

### Limitations

As with most research studies, the current study has many limitations, with some of the most relevant being the following. The recruitment process was extended to 11 months due to the heightened risk of fraud resulting from false identification of participants. This limitation was mitigated by excluding participants who did not complete sociodemographic questions and did not respond to the survey within a reasonable period. The study was underway at the beginning of the COVID-19 pandemic. Survey participants may have been approached more frequently within a short period, leading them to produce suboptimal responses ((49)). The limited information gathered on study participants and the high percentage of missing information could affect the validity of the results of the study. To mitigate this bias, careful selection of statistical methods that minimize the impact of missing data, such as including the valid percentage in the analysis, were carried out. Results were derived from a small sample of Facebook users, therefore, generalizability should be contextualized accordingly.

## Conclusion

The results of the current study suggest that infographics can be an important social marketing component for educating parents about the vaccination of school-aged children against HPV. These visual representations should focus on vaccine safety to respond to the information needs of parents. However, focusing this intervention on parents may not increase vaccine uptake ((50)). Infographics are more effective in increasing vaccine uptake in combination with other strategies, such as clear and accurate healthcare provider recommendations ((50–52)). Healthcare providers should also know about common misinformation/disinformation circulated in social media and be prepared to address this through appropriate educational techniques, such as attitudinal inoculation ((53)). Further research into how to use social marketing approaches to address such misinformation/disinformation may provide valuable insights on how to improve vaccination rates among school-aged children.

## Appendix

Supplemental material is available upon request to the author: mtantcho@uoguelph.ca
